# Impact of coronary collateralization on major adverse cardiovascular and cerebrovascular events after successful recanalization of chronic total occlusion

**DOI:** 10.3389/fcvm.2024.1374398

**Published:** 2024-06-25

**Authors:** Yurong Sun, Bin Zhang, Xinyuan Zhang, Xiaojiao Zhang, Wenqi Bao, Hangrui Bai, Bo Luan

**Affiliations:** ^1^ Internal Medicine, Dalian Medical University, Dalian, China; ^2^ Department of Cardiology, People’s Hospital of Liaoning Province, The People’s Hospital of China Medical University, Shenyang, Liaoning, China; ^3^ Clinical Medicine, China Medical University, Shenyang, Liaoning, China

**Keywords:** chronic total occlusion, metabolic syndrome, coronary collateral circulation, prognosis, major adverse cardiovascular and cerebrovascular events (MACCEs)

## Abstract

**Aims:**

This study aims to investigate the effects of coronary collateral circulation (CCC) on the prognosis of chronic total occlusion (CTO) patients with or without metabolic syndrome (MetS).

**Methods:**

The study included 342 CTO patients who underwent successful percutaneous coronary intervention at the People's Hospital of Liaoning Province between 1 February 2021 and 30 September 2023. The Rentrop score was used to assess the status of CCC. The outcome was major adverse cardiovascular and cerebrovascular events (MACCEs), defined as a composite of all-cause mortality, cardiac death, non-fatal myocardial infarction (MI), target vessel revascularization (TVR), and non-fatal stroke. Univariate and multivariate logistic analyses were used to investigate the association of CCC, MetS, and MACCEs with odds ratios (ORs) and 95% confidence intervals (CIs). The effect of CCC was further investigated in different MetS, diabetes mellitus (DM), and Syntax score groups.

**Results:**

MACCEs were more common in patients with poor CCC compared to those with good CCC (38.74% vs. 16.56%). Statistical differences were found in MACCEs (OR = 3.33, 95% CI: 1.93–5.72), MI (OR = 3.11, 95% CI: 1.73–5.58), TVR (OR = 3.06, 95% CI: 1.70–5.53), and stent thrombosis (OR = 6.14, 95% CI: 2.76–13.65) between the good and poor CCC groups. Poor CCC patients with MetS had a higher incidence of MACCEs (OR = 4.21, 95% CI: 2.05–8.65), non-fatal MI (OR = 4.44, 95% CI: 2.01–9.83), TVR (OR = 3.28, 95% CI: 1.51–7.11), and stent thrombosis (OR = 10.80, 95% CI: 3.11–37.54). Similar findings were also observed in CTO patients with DM and a Syntax score ≥23.

**Conclusion:**

Poor CCC could increase the risk of MACCEs in CTO patients, particularly those with MetS, DM, and a Syntax score ≥23. Further prospective, multicenter studies are needed to validate our findings and to explore potential therapeutic interventions.

## Introduction

Chronic total occlusion (CTO) is a common occurrence in patients with coronary artery disease (CAD), affecting a third of patients with CAD (1, 2). The main treatment for CTO is percutaneous coronary intervention (PCI) to achieve revascularization. Previous studies have shown several clinical benefits of successful CTO recanalization, including angina relief, decreased ischemic burden, and even increased survival (3). Coronary collateral circulation (CCC) plays a vital role in maintaining myocardial perfusion in the presence of coronary artery occlusion (4). Previous studies have suggested that well-developed collaterals could reduce infarct size and improve ventricular function, benefit CTO-PCI revascularization, and be related to a better long-term prognosis in patients with CAD (4, 5).

Metabolic syndrome (MetS), a disease related to multiple factors, could lead to a poor prognosis for cardiovascular disease (6, 7). It has been reported that diabetic patients with CTO are associated with a higher incidence of revascularization and total adverse cardiovascular events over a period of 5 years (8). Successful CTO revascularization in diabetic patients may be related to better long-term survival benefits, but this is not observed in the non-diabetic population (9–11). Yilmaz et al. (12) found that the incidence of MetS was higher in patients with poor circulation compared to those with good CCC. As MetS is similar to diabetes, we speculate that poor CCC and MetS may also adversely affect the long-term clinical prognosis of CTO patients after PCI.

In this study, we aimed to investigate the effect of CCC on major adverse cardiovascular and cerebrovascular events (MACCEs) in patients with and without MetS after successful CTO-PCI. The findings from this study may have important implications for risk stratification and treatment strategies for patients with CTO-PCI.

## Methods

### Study population

This is a retrospective cohort study conducted at the People's Hospital of Liaoning Province between February 2021 and September 2023. The inclusion criteria were as follows: (1) aged ≥18 years, (2) diagnosed with CTO, (3) without a history of PCI or coronary artery bypass grafting (CABG), and (4) with complete clinical data. Patients were excluded based on at least one of the following conditions: (1) contraindications for PCI or contrast agent injection; (2) concurrent cardiac diseases like heart failure or pulmonary heart disease; (3) severely impaired liver or kidney functions; and (4) malignant tumors or immune system diseases.

CTO was defined as arteries occluded for a documented duration of occlusion ≥3 months with absolutely antegrade flow through the lesion [thrombolysis in myocardial infarction (TIMI) grade 0 flow] (13). The Syntax score served as a reproducible angiographic tool to quantify the extent of coronary artery disease. MetS was determined based on the criteria of the International Diabetes Federation (14). Participants were required to have a waist circumference of ≥94 cm (men) or ≥80 cm (women). Meanwhile, participants needed to meet at least two of the following criteria: (1) glucose levels ≥5.6 mmol/L or diagnosed diabetes; (2) low high-density lipoprotein cholesterol (HDL-C) levels <1.0 mmol/L (men), <1.3 mmol/L (women), or receiving drug treatment for low HDL-C; (3) triglyceride (TG) levels ≥1.7 mmol/L or receiving drug treatment for high TG; (4) blood pressure ≥130/85 mmHg or receiving drug treatment for hypertension. The study protocol was reviewed and approved by the Ethics Committee of the People's Hospital of Liaoning Province (Approval No. 2023-K063). All participants signed written informed consent.

### Data collection

Trained physicians or nurses collected the following information about patients: demographic data, disease characteristics, treatment-related data, and occurrences of MACCEs. Demographic data included age, gender, height, weight, smoking and drinking habits, family history of CAD, history of myocardial infarction (MI), cerebral infarction, diabetes mellitus (DM), and MetS. Before the coronary interventions were performed, information on the characteristics of the disease was collected, including the number of occluded vessels, location of the CTO lesion, left ventricular ejection fraction, number of recanalized vessels in the CTO lesion, complete revascularization, and number of implanted stents. Treatment-related data included the type of therapeutic drugs, such as angiotensin-converting enzyme inhibitors (ACEIs) or angiotensin receptor blockers (ARBs), β-blockers, statins, and hypoglycemic drugs.

### Assessment of collateral circulation

The Rentrop scoring system was used to evaluate the grading of coronary collateralization: grade 0 indicates no visible filling of any collateral vessel, grade 1 indicates filling of the side branches by collateral vessels without filling of the epicardial arteries, grade 2 indicates partial filling of the epicardial artery by collateral vessels, and grade 3 indicates complete filling of the epicardial artery by collateral vessels (15). The Rentrop classification, categorized as grade 0 or 1, was defined as a poor coronary collateralization group, and grade 2 or 3 was considered a good group.

### Outcomes and follow-up

The outcome was MACCEs, consisting of all-cause death, cardiac death, non-fatal MI, target vessel revascularization (TVR), and non-fatal stroke (15). Cardiac death was defined as any death for which a definite non-cardiac cause could not be determined. MI was defined as participants with typical chest pain, ST-segment deviation, T wave changes, and creatine kinase-myocardial band levels at least three times the upper limit of normal (16). TVR, which included interventions on the target and non-target vessels by PCI or CABG, was performed in patients with severe in-stent restenosis or newly emerged coronary lesions (70% luminal diameter stenosis) (15). The study population was followed up at 3, 6, 9, and 12 months after discharge through office interviews, outpatient visits, telephone consultations, and a review of medical records.

### Statistical analysis

The normality of continuous variables was tested by skewness and kurtosis, while homogeneity was detected by the Levene test. Continuous variables with a normal distribution were described by the mean ± SD (standard deviation), while variables without a normal distribution were described by the median (interquartile range). Categorical variables were expressed as numbers and percentages. Student's *t*-test was used to compare group differences for continuous variables satisfying normal distribution and homogeneity of variance. A Satterthwaite *t*-test was used for continuous variables exhibiting normal distribution but lacking homogeneity of variance. For continuous variables that did not exhibit a normal distribution or homogeneity of variance, the Wilcoxon rank-sum test was used to evaluate differences between the two groups. The chi-squared test and Fisher's exact test were conducted to assess categorical variables between different groups, while the Wilcoxon rank-sum test was used for rank data. Covariates with *P* < 0.05 on univariate logistic analysis were considered potential confounders. Multivariable logistic regression analyses were conducted to investigate the relationship between the status of CCC and MACCEs. The results were presented as odds ratios (ORs) with their corresponding 95% confidence intervals (CIs). Survival curves were plotted for the two groups using the Kaplan–Meier method. Subgroup analyses stratified by MetS were also performed to explore the association between CCC and MACCEs. Model 1 was the crude model. Model 2 was adjusted for history of MI, number of occluded vessels, ACEI or ARB, and statins. The association of CCC with MACCEs was also explored in different DM and Syntax score subgroups. A two-sided *P* < 0.05 was used to indicate statistical significance. All analyses were performed using R version 4.2.3 (2023-03-15 ucrt).

## Results

### Characteristics of CTP patients

**Figure 1:**
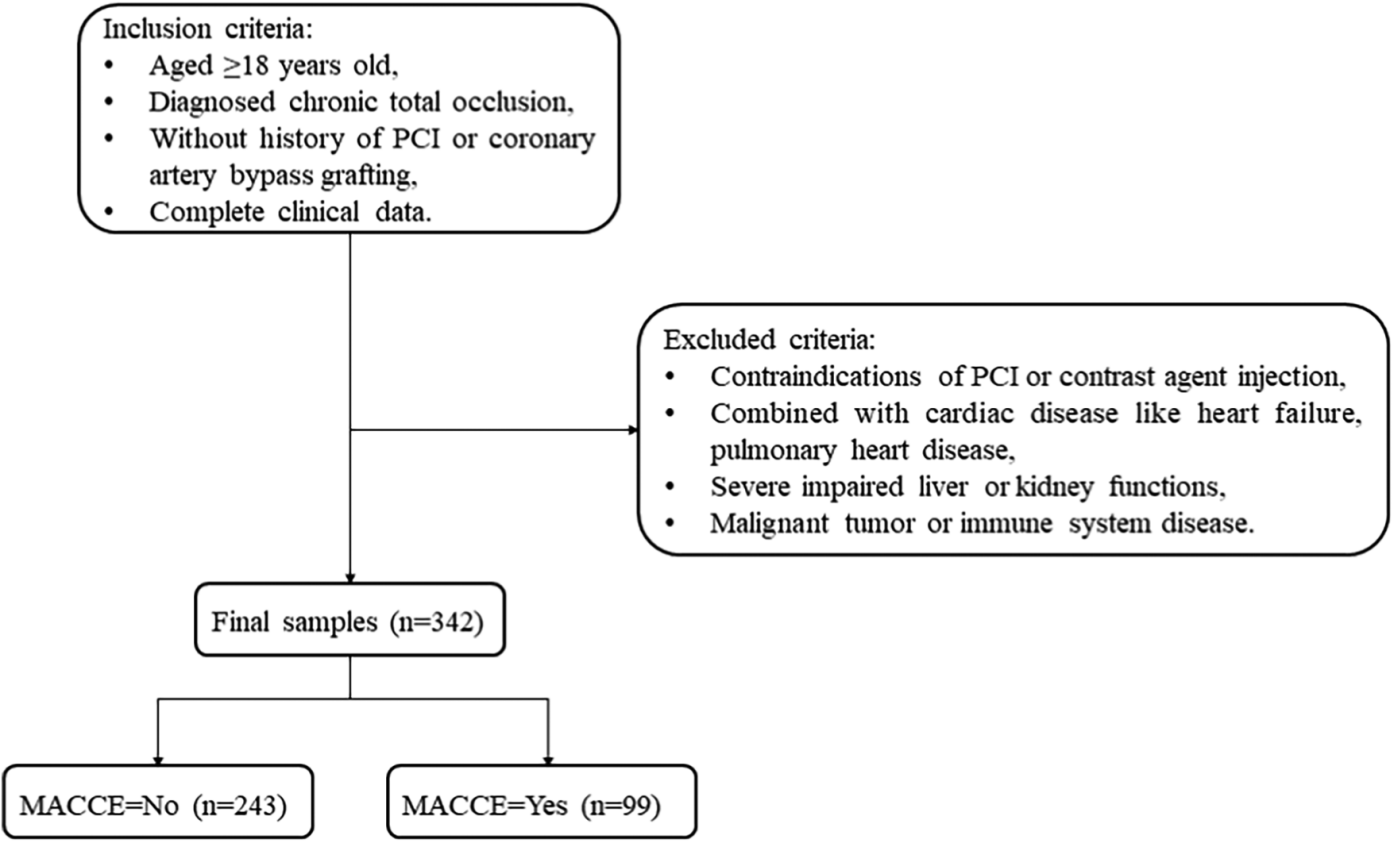
Flowchart of CTO patient screening.

A total of 342 CTO patients undergoing PCI were enrolled, with an average age of 61.43 years. Among them, 151 patients were classified as having a good CCC. There was statistical significance between the two groups in terms of smoking (*P* < 0.05). The demographic, clinical, and treatment information is presented in Table 1. Figure 1 illustrates the participants selection process.

**Table 1:** Characteristics of CTO patients with good and poor CCC.

Variables	Total (*N* = 342)	Good CCC (*N* = 151)	Poor CCC (*N* = 191)	Statistics	*P*
Age, mean (±SD)	61.43 (±10.51)	61.68 (±10.24)	61.23 (±10.75)	t = 0.394	0.694
Sex, *n* (%)				*χ*^2^ = 0.424	0.515
Male	265 (77.49)	120 (79.47)	145 (75.92)		
Female	77 (22.51)	31 (20.53)	46 (24.08)		
Height, m, mean (±SD)	1.69 (±0.07)	1.69 (±0.07)	1.69 (±0.07)	t = −0.502	0.616
Weight, kg, mean (±SD)	73.20 (±11.87)	73.26 (±10.75)	73.16 (±12.72)	t’ = 0.085	0.932
BMI, kg/m^2^, mean (±SD)	25.52 (±3.40)	25.60 (±2.94)	25.46 (±3.73)	t’ = 0.408	0.684
Smoking, *n* (%)				χ^2^ = 7.950	0.019
Never smoker	67 (19.59)	25 (16.56)	42 (21.99)		
Former smoker	162 (47.37)	64 (42.38)	98 (51.31)		
Current smoker	113 (33.04)	62 (41.06)	51 (26.7)		
Drinking, *n* (%)				χ^2^ = 1.534	0.464
Never drinker	62 (18.13)	24 (15.89)	38 (19.9)		
Former drinker	249 (72.81)	115 (76.16)	134 (70.16)		
Current drinker	31 (9.06)	12 (7.95)	19 (9.95)		
Family history of coronary artery disease, *n* (%)				χ^2^ = 0.085	0.771
No	326 (95.32)	145 (96.03)	181 (94.76)		
Yes	16 (4.68)	6 (3.97)	10 (5.24)		
History of myocardial infarction, *n* (%)				χ^2^ = 0.000	1.000
No	146 (42.69)	64 (42.38)	82 (42.93)		
Yes	196 (57.31)	87 (57.62)	109 (57.07)		
Cerebral infarction, *n* (%)				χ^2^ = 0.072	0.788
No	293 (85.67)	128 (84.77)	165 (86.39)		
Yes	49 (14.33)	23 (15.23)	26 (13.61)		
MetS, *n* (%)				χ^2^ = 2.348	0.125
No	162 (47.37)	64 (42.38)	98 (51.31)		
Yes	180 (52.63)	87 (57.62)	93 (48.69)		
Survival time, day, M (Q_1_, Q_3_)	365.00 (315.00, 366.00)	365.00 (365.00,365.00)	365.00 (257.00, 366.00)	W = 15,704.500	0.138
Syntax score, mean (±SD)	18.93 (±7.78)	17.98 (±7.59)	19.68 (±7.87)	t = −2.014	0.045
LVEF, %, mean (±SD)	44.01 (±6.75)	44.72 (±6.57)	43.46 (±6.85)	t = 1.728	0.085
Number of recanalized vessels in the CTO lesion, mean (±SD)	1.10 (±0.34)	1.09 (±0.31)	1.10 (±0.35)	t = −0.327	0.744
Number of occluded vessels, *n* (%)				χ^2^ = 0.047	0.828
1	294 (85.96)	131 (86.75)	163 (85.34)		
2 or 3	48 (14.04)	20 (13.25)	28 (14.66)		
Location of the CTO lesion					
Left anterior descending, *n* (%)				χ^2^ = 0.500	0.480
No	216 (63.16)	99 (65.56)	117 (61.26)		
Yes	126 (36.84)	52 (34.44)	74 (38.74)		
Left circumflex artery, *n* (%)				χ^2^ = 0.374	0.541
No	270 (78.95)	122 (80.79)	148 (77.49)		
Yes	72 (21.05)	29 (19.21)	43 (22.51)		
Right coronary artery, *n* (%)				χ^2^ = 0.560	0.454
No	147 (42.98)	61 (40.4)	86 (45.03)		
Yes	195 (57.02)	90 (59.6)	105 (54.97)		
Number of stents for the CTO vessel, mean (±SD)	2.26 (±1.18)	2.23 (±1.21)	2.28 (±1.15)	t = −0.356	0.722
Complete revascularization, *n* (%)				−	0.633
No	4 (1.17)	1 (0.66)	3 (1.57)		
Yes	338 (98.83)	150 (99.34)	188 (98.43)		
ACEIs or ARBs, *n* (%)				χ^2^ = 0.380	0.537
No	51 (14.91)	20 (13.25)	31 (16.23)		
Yes	291 (85.09)	131 (86.75)	160 (83.77)		
β-blockers, *n* (%)				χ^2^ = 0.421	0.516
No	42 (12.28)	21 (13.91)	21 (10.99)		
Yes	300 (87.72)	130 (86.09)	170 (89.01)		
Statins, *n* (%)				χ^2^ = 0.061	0.805
No	39 (11.4)	16 (10.6)	23 (12.04)		
Yes	303 (88.6)	135 (89.4)	168 (87.96)		
Hypoglycemic drugs, *n* (%)				χ^2^ = 3.779	0.052
No	194 (56.73)	95 (62.91)	99 (51.83)		
Yes	148 (43.27)	56 (37.09)	92 (48.17)		

SD, standard deviation; t, Student's *t*-test; t', Satterthwaite *t*-test; χ^2^, chi-squared test; -, Fisher's exact test; W, Wilcoxon rank-sum test; BMI, body mass index; LVEF, left ventricular ejection fraction.

### MACCEs in CTO patients

**Figure 2:**
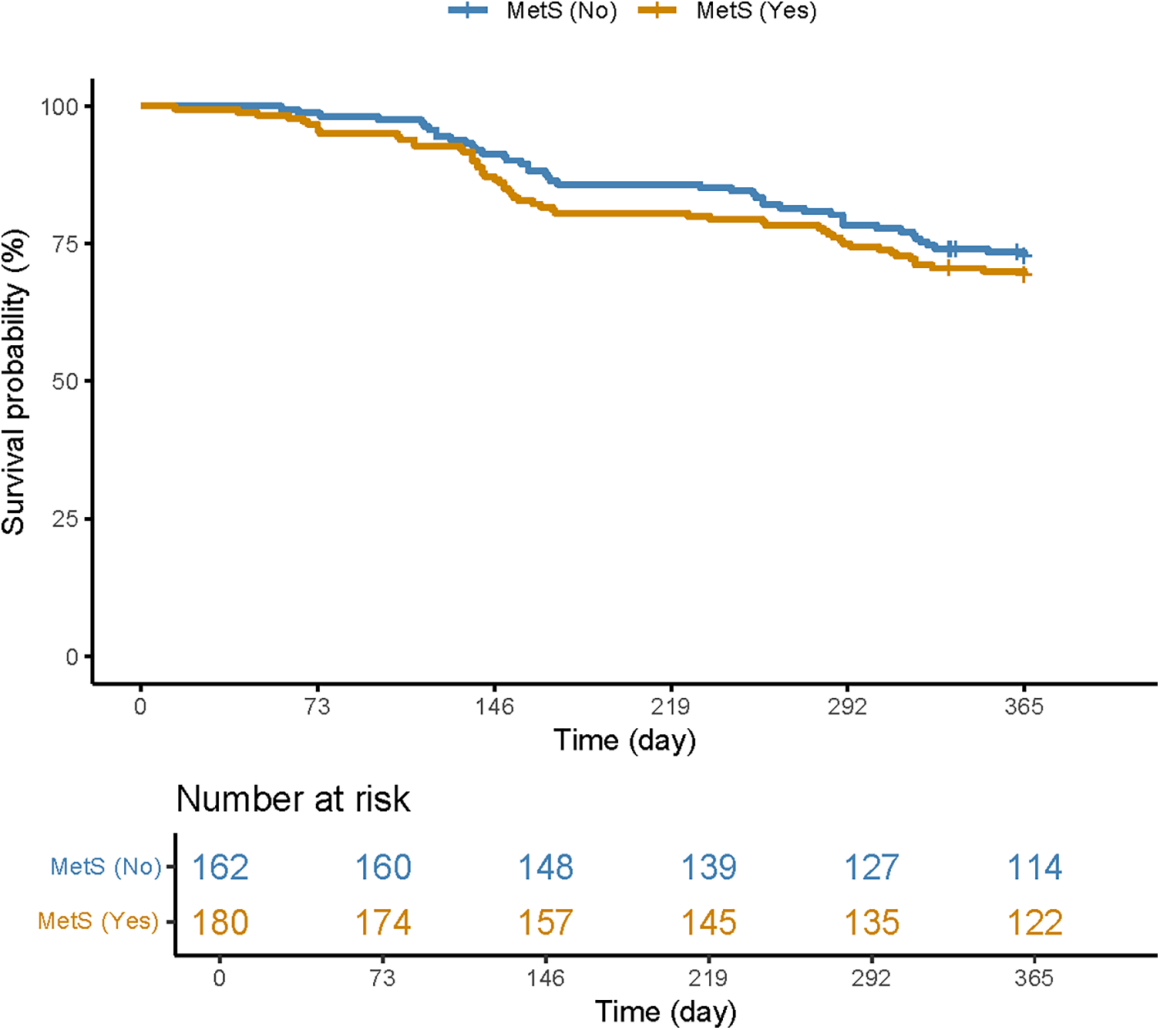
Kaplan–Meier curve plotted for patients with and without MetS.

Table 2 presents the clinical outcomes of CTO patients with good or poor CCC. During the 1-year follow-up period, 99 CTO patients experienced MACCEs. In total, 18 CTO patients succumbed to all-cause death, with 17 of them being attributed to cardiac death. In addition, 78 CTO patients experienced non-fatal MI, while 15 CTO patients suffered a non-fatal stroke. The survival curve of the MetS group was significantly lower than that of the non-MetS group (Figure 2). All participants received coronary angiography during follow-up, with 77 of them undergoing repeat revascularization. Overall, the rate of MACCEs and their components was higher in patients with poor CCC compared to those with good CCC.

**Table 2:** Clinical outcomes of CTO patients with good or poor CCC.

Variables	Total (*N* = 342)	Good CCC (*N* = 151)	Poor CCC (*N* = 191)	Statistics	*P*
MACCEs, *n* (%)				χ^2^ = 19.119	<0.001
No	243 (71.05)	126 (83.44)	117 (61.26)		
Yes	99 (28.95)	25 (16.56)	74 (38.74)		
All-cause death, *n* (%)				χ^2^ = 0.498	0.480
No	324 (94.74)	145 (96.03)	179 (93.72)		
Yes	18 (5.26)	6 (3.97)	12 (6.28)		
Cardiac death, *n* (%)				χ^2^ = 1.010	0.315
No	325 (95.03)	146 (96.69)	179 (93.72)		
Yes	17 (4.97)	5 (3.31)	12 (6.28)		
Non-fatal MI, *n* (%)				χ^2^ = 15.031	<0.001
No	264 (77.19)	132 (87.42)	132 (69.11)		
Yes	78 (22.81)	19 (12.58)	59 (30.89)		
TVR, *n* (%)				χ^2^ = 14.285	<0.001
No	265 (77.49)	132 (87.42)	133 (69.63)		
Yes	77 (22.51)	19 (12.58)	58 (30.37)		
Non-fatal stroke, *n* (%)				χ^2^ = 0.356	0.550
No	327 (95.61)	146 (96.69)	181 (94.76)		
Yes	15 (4.39)	5 (3.31)	10 (5.24)		
Stent thrombosis, *n* (%)				χ^2^ = 22.798	<0.001
No	286 (83.63)	143 (94.7)	143 (74.87)		
Yes	56 (16.37)	8 (5.3)	48 (25.13)		

χ^2^: chi-squared test.

### Association between CCC and MACCEs in CTO patients

In model 2, confounders were adjusted, including history of MI, number of occluded vessels, ACEI or ARB, and statin use. Poor CCC was related to a higher incidence of MACCEs (OR = 3.33, 95% CI: 1.93–5.72), non-fatal MI (OR = 3.11, 95% CI: 1.73–5.58), TVR (OR = 3.06, 95% CI: 1.70–5.53), and stent thrombosis (OR = 6.14, 95% CI: 2.76–13.65) (Table 3).

**Table 3:** Association of CCC with MACCEs in CTO patients.

Variables	Outcome/total (*n*)	Model 1		Model 2	
		OR (95% CI)	*P*	OR (95% CI)	*P*
MACCEs					
Good CCC	25/151	Ref		Ref	
Poor CCC	74/191	3.19 (1.90–5.35)	<0.001	3.33 (1.93–5.72)	<0.001
All-cause death					
Good CCC	6/151	Ref		Ref	
Poor CCC	12/191	1.62 (0.59–4.42)	0.346	1.52 (0.55–4.23)	0.421
Cardiac death					
Good CCC	5/151	Ref		Ref	
Poor CCC	12/191	1.96 (0.67–5.68)	0.217	1.82 (0.62–5.37)	0.276
Non-fatal MI					
Good CCC	19/151	Ref		Ref	
Poor CCC	59/191	3.11 (1.76–5.49)	<0.001	3.11 (1.73–5.58)	<0.001
TVR					
Good CCC	19/151	Ref		Ref	
Poor CCC	58/191	3.03 (1.71–5.36)	<0.001	3.06 (1.70–5.53)	<0.001
Non-fatal stroke					
Good CCC	5/151	Ref		Ref	
Poor CCC	10/191	1.61 (0.54–4.82)	0.392	1.59 (0.53–4.77)	0.412
Stent thrombosis					
Good CCC	8/151	Ref		Ref	
Poor CCC	48/191	6.00 (2.74–13.14)	<0.001	6.14 (2.76–13.65)	<0.001

Ref, reference. Model 1: crude model. Model 2: adjusted for MI history, the number of occluded vessels, ACEIs or ARBs, and statin use.

The relationship of CCC status with MACCEs was further assessed in CTO patients with or without MetS. Poor CCC in patients with MetS was associated with higher odds of MACCEs (OR = 4.21, 95% CI: 2.05–8.65), non-fatal MI (OR = 4.44, 95% CI: 2.01–9.83), TVR (OR = 3.28, 95% CI: 1.51–7.11), and stent thrombosis (OR = 10.80, 95% CI: 3.11–37.54) (Table 4).

**Table 4:** Association of coronary collateral circulation with MACCEs and their components in patients with or without MetS.

Subgroups	Outcome/total	Model 1		Model 2	
		OR (95% CI)^a^	*P*	OR (95% CI)^a^	*P*
Non-MetS					
MACCEs	*N* = 33/98	2.45 (1.13–5.30)	0.023	2.54 (1.14–5.64)	0.022
All-cause mortality	*N* = 6/98	1.33 (0.32–5.50)	0.697	1.34 (0.31–5.80)	0.692
Cardiac death	*N* = 6/98	1.33 (0.32–5.50)	0.697	1.34 (0.31–5.80)	0.692
Non-fatal MI	*N* = 25/98	2.09 (0.90–4.84)	0.084	2.09 (0.90–4.85)	0.087
TVR	*N* = 28/98	2.80 (1.18–6.62)	0.019	2.93 (1.20–7.13)	0.018
Non-fatal stroke	*N* = 5/98	1.67 (0.31–8.86)	0.549	1.66 (0.31–8.86)	0.551
Stent thrombosis	*N* = 22/98	3.42 (1.22–9.56)	0.019	3.45 (1.22–9.76)	0.020
MetS					
MACCEs	*N* = 41/93	4.11 (2.03–8.31)	<0.001	4.21 (2.05–8.65)	<0.001
All-cause mortality	*N* = 6/93	1.93 (0.47–7.97)	0.363	1.97 (0.47–8.26)	0.354
Cardiac death	*N* = 6/93	2.93 (0.58–14.93)	0.195	2.92 (0.57–14.92)	0.197
Non-fatal MI	*N* = 34/93	4.44 (2.03–9.70)	<0.001	4.44 (2.01–9.83)	<0.001
TVR	*N* = 30/93	3.29 (1.53–7.09)	0.002	3.28 (1.51–7.11)	0.003
Non-fatal stroke	*N* = 5/93	1.59 (0.37–6.87)	0.534	1.60 (0.36–7.05)	0.532
Stent thrombosis	*N* = 26/93	10.87 (3.15–37.45)	<0.001	10.80 (3.11–37.54)	<0.001

Model 1: crude model. Model 2: adjusted for myocardial infarction and the number of occluded vessels.

^a^ Good CCC as the reference.

### Association between CCC and MACCEs in CTO patients with different DM and Syntax score subgroups

Table 5 illustrates the relationship between CCC and MACCEs in different DM and Syntax score subgroups. In DM patients, poor CCC was related to higher odds of MACCEs (OR = 4.42, 95% CI: 1.96–10.97), non-fatal MI (OR = 4.12, 95% CI: 1.70–11.39), TVR (OR = 3.09, 95% CI: 1.34–7.83), and stent thrombosis (OR = 10.98, 95% CI: 2.97–71.98). In CTO patients with Syntax score ≥23, poor CCC was associated with a higher incidence of MACCEs (OR = 3.83, 95% CI: 1.43–11.72), non-fatal MI (OR = 5.89, 95% CI: 1.77–27.28), TVR (OR = 3.45, 95% CI: 1.19–11.89), and stent thrombosis (OR = 11.49, 95% CI: 2.64–89.60).

**Table 5:** Association of coronary collateral circulation with MACCEs in different DM and syntax score subgroups.

Subgroups	Outcome/total	Model 1		Model 2	
		OR (95% CI)^a^	*P*	OR (95% CI)^a^	*P*
DM					
MACCEs	*N* = 36/94	3.60 (1.69–8.28)	0.001	4.42 (1.96–10.97)	0.001
All-cause mortality	*N* = 6/94	2.25 (0.50–15.69)	0.330	2.81 (0.58–21.12)	0.237
Cardiac death	*N* = 6/94	4.57 (0.76–87.37)	0.164	5.25 (0.84–102.25)	0.134
Non-fatal MI	*N* = 27/94	3.51 (1.50–9.28)	0.006	4.12 (1.70–11.39)	0.003
TVR	*N* = 27/94	2.64 (1.19–6.37)	0.022	3.09 (1.34–7.83)	0.012
Non-fatal stroke	*N* = 5/94	1.85 (0.39–13.22)	0.469	1.86 (0.38–13.39)	0.470
Stent thrombosis	*N* = 21/94	9.49 (2.65–60.79)	0.003	10.98 (2.97–71.98)	0.002
Non-DM					
MACCEs	*N* = 38/97	2.92 (1.49–5.97)	0.002	2.89 (1.44–6.05)	0.004
All-cause mortality	*N* = 6/97	1.30 (0.36–5.25)	0.691	1.14 (0.30–4.70)	0.844
Cardiac death	*N* = 6/97	1.30 (0.36–5.25)	0.691	1.14 (0.30–4.70)	0.844
Non-fatal MI	*N* = 32/97	2.91 (1.42–6.33)	0.005	2.86 (1.37–6.30)	0.006
TVR	*N* = 31/97	3.43 (1.61–7.86)	0.002	3.30 (1.53–7.65)	0.003
Non-fatal stroke	*N* = 5/97	1.45 (0.34–7.24)	0.619	1.55 (0.36–7.86)	0.561
Stent thrombosis	*N* = 27/97	4.95 (2.05–13.90)	0.001	4.83 (1.97–13.74)	0.001
Syntax score <23					
MACCEs	*N* = 50/134	3.01 (1.67–5.61)	<0.001	2.97 (1.62–5.64)	0.001
All-cause mortality	*N* = 6/134	1.75 (0.45–8.44)	0.436	1.64 (0.41–8.01)	0.500
Cardiac death	*N* = 7/134	2.06 (0.56–9.72)	0.304	1.94 (0.52–9.25)	0.350
Non-fatal MI	*N* = 40/134	2.63 (1.40–5.14)	0.003	2.53 (1.34–5.00)	0.005
TVR	*N* = 39/134	2.96 (1.54–5.97)	0.002	2.86 (1.47–5.82)	0.003
Non-fatal stroke	*N* = 18/57	7.85 (2.06–51.64)	0.008	11.49 (2.64–89.60)	0.005
Stent thrombosis	*N* = 30/134	5.24 (2.23–14.42)	<0.001	5.52 (2.30–15.49)	<0.001
Syntax score ≥23					
MACCEs	*N* = 24/57	3.64 (1.37–10.91)	0.013	3.83 (1.43–11.72)	0.011
All-cause mortality	*N* = 6/57	1.29 (0.32–6.46)	0.728	1.31 (0.32–6.70)	0.717
Cardiac death	*N* = 5/57	1.63 (0.33–11.87)	0.570	1.83 (0.35–14.12)	0.502
Non-fatal MI	*N* = 19/57	5.50 (1.69–24.90)	0.010	5.89 (1.77–27.28)	0.009
TVR	*N* = 19/57	3.10 (1.10–10.21)	0.043	3.45 (1.19–11.89)	0.032
Non-fatal stroke	*N* = 2/57	1.27 (0.12–27.99)	0.846	1.37 (0.12–31.04)	0.805
Stent thrombosis	*N* = 18/57	7.85 (2.06–51.64)	0.008	11.49 (2.64–89.60)	0.005

Model 1: crude model. Model 2: adjusted for myocardial infarction, the number of occluded vessels, ACEIs or ARBs, and statins for the DM subgroup. Adjusted for myocardial infarction, ACEIs or ARBs, and statins for the Syntax score.

^a^ Good CCC as the reference.

## Discussion

Our study investigated the relationship between CCC and MACCEs in patients who underwent PCI for CTO. The results suggested that poor CCC was associated with MACCEs, non-fatal MI, TVR, and stent thrombosis in CTO patients. Similar findings were observed in CTO patients with MetS, with even significantly higher odds of MACCEs. This suggests that the CCC status of CTO patients and MetS may have a combined effect on MACCEs.

Our findings were consistent with previous studies on the impact of CCC status on the prognosis of CTO patients (15, 17, 18). CCC is a beneficial prognostic factor (19). Collateral vessels provide an important alternative route for blood flow, especially in vessel occlusion, and are associated with improved outcomes and reduced ischemic injury (20). Conversely, poor collateralization has been related to adverse events such as myocardial infarction and mortality (21). In contrast, Li et al. (22) reported that good CCC was not associated with a lower risk of cardiac death or MACCEs in CTO patients. Some factors, such as coronary steal, microcirculation dilation, and endothelial dysfunction, may offset the potential benefits of collateral vessels, thus leading to inadequate oxygen and flow supply through collateral vessels, concomitant with an elevated predisposition to arrhythmias in patients with good CCC. Future studies are needed to clarify the relationship between CCC and MACCEs in CTO patients.

In addition, the impact of poor CCC on MACCEs was particularly pronounced in patients with MetS. MetS constitutes a constellation of risk factors, including central obesity, insulin resistance, hypertension, and dyslipidemia, and is associated with poor coronary collateralization and increased cardiovascular risk (6, 23). Our results revealed that poor CCC in CTO patients with MetS was associated with higher odds of MACCEs and related events.

The mechanisms underlying the association between poor CCC and MACCEs in CTO patients with MetS involve complex pathophysiological interactions. In patients undergoing CTO-PCI, poor CCC may reflect a higher burden of coronary artery burden, with impaired development of collateral vessels unable to sufficiently compensate for the occluded vessel (24). This impaired collateralization may result from genetic predisposition, microvascular dysfunction, or inadequate release of pro-angiogenic factors (25, 26). Inadequate collateral support resulted in ongoing myocardial ischemia, impaired myocardial function, and increased susceptibility to adverse events (27, 28). Furthermore, consistent with our results, stent thrombosis was more prevalent in poor CCC, as collateral flow has been shown to protect against thrombus formation and facilitate myocardial reperfusion (29).

In patients with MetS, poor CCC may further exacerbate the cardiovascular effects associated with the syndrome. The presence of MetS is associated with endothelial dysfunction, chronic inflammation, and a prothrombotic state, all of which may contribute to impaired collateral vessel formation and function (30, 31). The presence of poor CCC in MetS patients may signify an inability to adequately respond to ischemic insults, leading to an increased risk of adverse events (32). There may be a combined effect between MetS and CCC.

In CTO patients with DM or a Syntax score ≥23, poor CCC was also related to high odds of MACCEs. It is well established that individuals with diabetes exhibit impaired collateral vessel development due to factors such as endothelial dysfunction, abnormal angiogenesis, and impaired growth factor signaling (33). These factors collectively contribute to reduced collateral vessel formation, resulting in compromised vascular supply to the ischemic myocardium. In the context of CTO patients with diabetes, this impaired collateralization may further aggravate the ischemic burden, leading to a higher risk of adverse cardiovascular events. A higher Syntax score indicates more severe and complex coronary artery disease, indicating the presence of multiple lesions or diffuse disease. In patients with a Syntax score ≥23, the extent of atherosclerotic burden is substantial, potentially leading to impaired collateral vessel formation and poorer perfusion to the myocardium. Moreover, the high complexity of lesions in this subgroup may increase the risk of stent thrombosis and TVR.

In patients who underwent CTO-PCI, the extent of CTO disease, blood glucose, blood lipids, and blood pressure should be monitored closely. Identifying patients with poor CCC following CTO-PCI and those with MetS could contribute to risk stratification in patients and guide targeted therapeutic interventions. More attention should be paid to CTO patients with DM and a Syntax score ≥23. Strategies aimed at enhancing CCC, such as physical activity, pharmacological interventions, and targeted revascularization strategies, may prove beneficial in these high-risk patient populations. In addition, close monitoring and aggressive management of modifiable risk factors may be warranted for individuals with poor CCC and MetS to mitigate their heightened risk of MACCEs and related events.

The current study has several limitations that need to be considered. First, the study population consisted of a single-center cohort, which may limit the generalizability of our findings. Multicenter, large-sample studies are needed in the future. Moreover, there were potential confounding factors that were not accounted for in this analysis, such as medication and calcification of blood vessels. Finally, clinical follow-up was relatively short, and the long-term prognostic relationship between CCC, MetS, and MACCEs was not fully investigated.

## Conclusion

Poor CCC has been associated with an increased risk of MACCEs in CTO patients, particularly those with MetS. Comprehensive risk evaluation and individualized management strategies are essential for patients with poor CCC. Further prospective multicenter studies are needed to confirm our results and investigate potential therapeutic interventions.

## Data Availability

The raw data supporting the conclusions of this article will be made available by the authors without undue reservation.
